# Genomic analysis of Salmonella Typhi from a typhoid conjugate vaccine trial

**DOI:** 10.1099/mgen.0.001721

**Published:** 2026-05-18

**Authors:** Belson M. Kutambe, Priyanka D. Patel, Kenneth Chizani, Niza Silungwe, Christopher Kukacha, Megan E. Carey, James E. Meiring, Matthew B. Laurens, Melita A. Gordon, Philip M. Ashton

**Affiliations:** 1 Malawi-Liverpool-Wellcome Programme, Blantyre, Malawi; 2 Institute of Infection, Veterinary and Ecological Sciences, University of Liverpool, Liverpool, UK; 3 Department of Infection Biology, London School of Hygiene & Tropical Medicine, London, UK; 4 MM Global Health, Zurich Switzerland and Amsterdam Institute for Global Health and Development, Amsterdam, The Netherlands; 5 Center for Vaccine Development and Global Health, University of Maryland School of Medicine, Baltimore, Maryland, USA; 6 The University of Edinburgh Usher Institute of Population Health Sciences and Informatics, Edinburgh, Scotland, UK; 7 Centre for Genomic Pathogen Surveillance, Pandemic Sciences Institute, University of Oxford, Oxford, UK

**Keywords:** *Salmonella *Typhi, typhoid conjugate vaccine, Vi antigen, whole-genome sequencing

## Abstract

**Background.** *Salmonella enterica* serovar Typhi (*S*. Typhi), the causative agent of typhoid fever, continues to pose a major public health challenge in many low- and middle-income countries. The World Health Organization recommended typhoid conjugate vaccine (TCV) use in countries with a high burden of disease and/or high rates of antimicrobial resistance (AMR). Recent introductions of TCVs into national immunization programmes are expected to substantially reduce this burden. However, the impact of vaccine introduction on pathogen populations has not been widely investigated.

**Methods.** To fill this research gap, we analysed the genomes of isolates from a trial in Malawi. A Phase 3, double-blind, randomized, controlled trial enrolled 28,130 healthy children aged 9 months through 12 years of age to receive either TCV (Typbar-TCV, Bharat Biotech) or meningococcal A conjugate vaccine (MenA). We conducted Illumina whole-genome sequencing and compared isolates from the TCV intervention arm to the MenA control arm with regard to (i) *S*. Typhi lineage distribution, (ii) AMR profile, (iii) mutations in genes associated with Vi capsule biosynthesis and expression and (iv) phylogenetic population structure.

**Results.** We obtained high-quality genome sequences for 136 *S*. Typhi isolates (24 from the TCV arm, 112 from the MenA arm). Of these, 135 (99%) belonged to lineage L4.3.1.1.EA1. Isolates from the two arms were intermixed across multiple sub-clades. Nearly all isolates (135 out of 136) carried genes encoding resistance to ampicillin, cotrimoxazole and chloramphenicol. Non-synonymous mutations in the quinolone resistance determining region were identified in five isolates (three GyrA S83F; two GyrA S464F), and no statistically significant difference in prevalence of these mutations was observed between study arms (Fisher’s exact test, *P*=0.25). Mutations in Vi capsule–associated genes were detected in 11 out of 112 (9%) MenA isolates and 1 out of 24 (4%) TCV isolates (Fisher’s exact test, *P*=0.67). Fifteen MenA cases were hospitalized, two of whom had Vi-associated mutations. In one of these cases, the Vi mutation has previously been associated with increased virulence in a murine model; however, there was no significant association between tested Vi mutations and hospitalization (*P*>0.91).

**Conclusions.** We identified no clinically meaningful genomic differences between *S*. Typhi isolates from participants vaccinated with TCV or MenA, and no Vi-negative *S*. Typhi were detected. These findings suggest no detectable short-term evolutionary impact of TCV on circulating *S*. Typhi populations in this Phase 3 trial. However, given the limited follow-up period and *S*. Typhi’s relatively slow substitution rate, continued genomic surveillance in this population after national TCV introduction in 2023 is essential to detect evolutionary responses to vaccine pressure.

**Impact Statement**

This study provides the first whole-genome comparison of *Salmonella* Typhi isolates collected from the intervention and control arms of a randomized Phase 3 typhoid conjugate vaccine (TCV) trial. By embedding pathogen genomics directly within a rigorously controlled vaccine efficacy study, we were able to test whether the evolutionary pressure exerted by the vaccine trial led to any detectable short-term evolutionary response in *S*. Typhi populations. We show that infections in vaccinated children are caused by the same dominant multidrug-resistant H58/L4.3.1.1.EA1 lineage circulating in the wider community, with no evidence of vaccine-associated clustering, shifts in antimicrobial resistance or enrichment of mutations in Vi-capsule biosynthesis and regulation. Importantly, we also find no evidence for the emergence of Vi-negative *S*. Typhi, a key theoretical route to vaccine escape. Beyond its specific findings for Malawi, this work demonstrates how whole-genome sequencing can be integrated into vaccine trials to provide population-level insight into pathogen response to immunological interventions. While post-introduction surveillance is increasingly used to monitor vaccine impact, randomized trial–based genomic comparisons remain rare. Our study establishes a genomic baseline for tracking the long-term evolutionary impact of TCV rollout and provides a scalable model for embedding pathogen genomics into the evaluation of vaccines against antimicrobial-resistant bacteria in low- and middle-income settings.

## Data Summary

The authors confirm that all supporting data, code and protocols have been provided within the article or through the supplementary data file. The raw sequencing reads for the 136 *S*. Typhi isolates subjected to whole-genome sequencing have been deposited in the European Nucleotide Archive under BioProject PRJEB104623 (see Table S1 for per isolate accession numbers).

## Introduction

Typhoid fever, caused by *Salmonella enterica* serovar Typhi (*S*. Typhi), remains a significant public health challenge, particularly in low-resource settings [1]. In recent decades, the emergence and spread of several multidrug-resistant [MDR (i.e. resistant to the first-line agents chloramphenicol, ampicillin and cotrimoxazole)] *S*. Typhi lineages, particularly H58 (clade 4.3.1) and H56 (clade 3.1.1), have complicated antimicrobial treatment and increased the urgency for effective prevention strategies [2]. Furthermore, *S*. Typhi can gain resistance to fluoroquinolones [2,3], third-generation cephalosporins [4] and azithromycin [5], raising concerns about the sustainability of antimicrobial therapy and highlighting the need for preventive interventions that can reduce both disease burden and antimicrobial use.

Typhoid conjugate vaccines (TCVs) represent a key public health tool to control typhoid fever and associated antimicrobial resistance (AMR), both by preventing drug-resistant *S*. Typhi infections and potentially reducing antimicrobial use at the population level [6]. These highly effective vaccines target the Vi capsular polysaccharide antigen and have been recently introduced in several high-burden countries [7]. While clinical trials and post-introduction studies have demonstrated strong protective efficacy [8,9], less is known about how widespread vaccine use may influence the evolutionary dynamics of circulating *S*. Typhi populations.

Evidence from other bacterial pathogens demonstrates that conjugate vaccines can substantially reshape pathogen population structure. Whole-genome sequencing studies of *Streptococcus pneumoniae* following pneumococcal conjugate vaccine introduction have shown declines in vaccine-targeted lineages accompanied by expansion of non-vaccine lineages, capsular switching and shifts in AMR profiles [10,17]. These findings highlight how vaccine-induced selective pressures can alter bacterial population composition over time and underscore the importance of genomic surveillance to monitor potential vaccine escape and evolutionary adaptation. Such studies have established population genomics as an important tool for understanding pathogen responses to vaccination beyond direct measurements of vaccine efficacy [13].

In contrast, genomic evidence describing population-level responses of *S*. Typhi to vaccination remains limited. Previous genomic analyses have characterized the global population structure and AMR evolution of *S*. Typhi [18,19], as well as genetic variation within the Vi capsular biosynthesis locus [20], suggesting that antigenic regions targeted by the TCV may accumulate evolutionary changes. Notably, Vi-negative *S*. Typhi isolates capable of causing disease have been reported in Pakistan [21], raising questions about the potential for vaccine-driven selection acting on Vi-associated genes. However, whether TCV introduction exerts measurable short-term evolutionary pressures on circulating *S*. Typhi populations in endemic settings has not yet been well characterized.

Monitoring pathogen population responses to vaccine pressure is therefore crucial to detect potential vaccine escape, lineage replacement or shifts in resistance profiles. In this study, we aimed to provide high-resolution insight into the short-term evolutionary dynamics of *S*. Typhi following TCV use by sequencing isolates collected during a Phase 3 vaccine trial in Malawi. We hypothesized that widespread TCV use may exert a selective pressure on *S*. Typhi populations potentially increasing the frequency of mutations in the Vi encoding genes among isolates from TCV recipients compared with those from the MenA control group. Furthermore, data collected during trial passive surveillance follow-up demonstrated that TCV use resulted in fewer antibiotic prescriptions [22]; hence, we hypothesized that lower transmission of *S*. Typhi and decreased selection pressure due to decreased antimicrobial use might result in a lower frequency of AMR genes in TCV recipients.

## Methods

### Sample collection and isolation

As part of a previously published vaccine trial, 28,130 children were vaccinated with either TCV (*n*=14,069) or MenA (*n*=14,061) [7,23]. If trial participants presented with febrile illness (subjective fever for ≥72 h, an axillary temperature of ≥38 °C or hospitalization with a history of fever of any duration), a blood culture was obtained (5 ml in children <5 years of age or 10 ml in children ≥5 years of age). AMR profiles of *S*. Typhi isolates were tested using disc diffusion. Isolates exhibiting pefloxacin resistance underwent confirmatory testing for ciprofloxacin resistance using E-test (bioMérieux), with a minimum inhibitory concentration of more than 0.06 mg per litre indicating non-susceptibility. Full details of study procedures can be found in [7,7, 23].

### Microbiology and molecular biology

Before sequencing, isolates were cultured overnight on XLD agar at 37 °C, and genomic DNA was extracted with the Wizard Genomic DNA Extraction Kit (Promega, Madison, WI, USA) following the manufacturer’s recommendations. The first batch of genomic DNA was shipped to the Wellcome Sanger Institute for indexed whole-genome sequencing on an Illumina HiSeq 2500 or HiSeq 4000 platform (Illumina, San Diego, CA, USA) to generate paired-end reads of 100 bp in length, and subsequent batches were sequenced at the Malawi Liverpool Wellcome Program on Illumina MiSeq.

### Bioinformatics

**Fig. 1.**
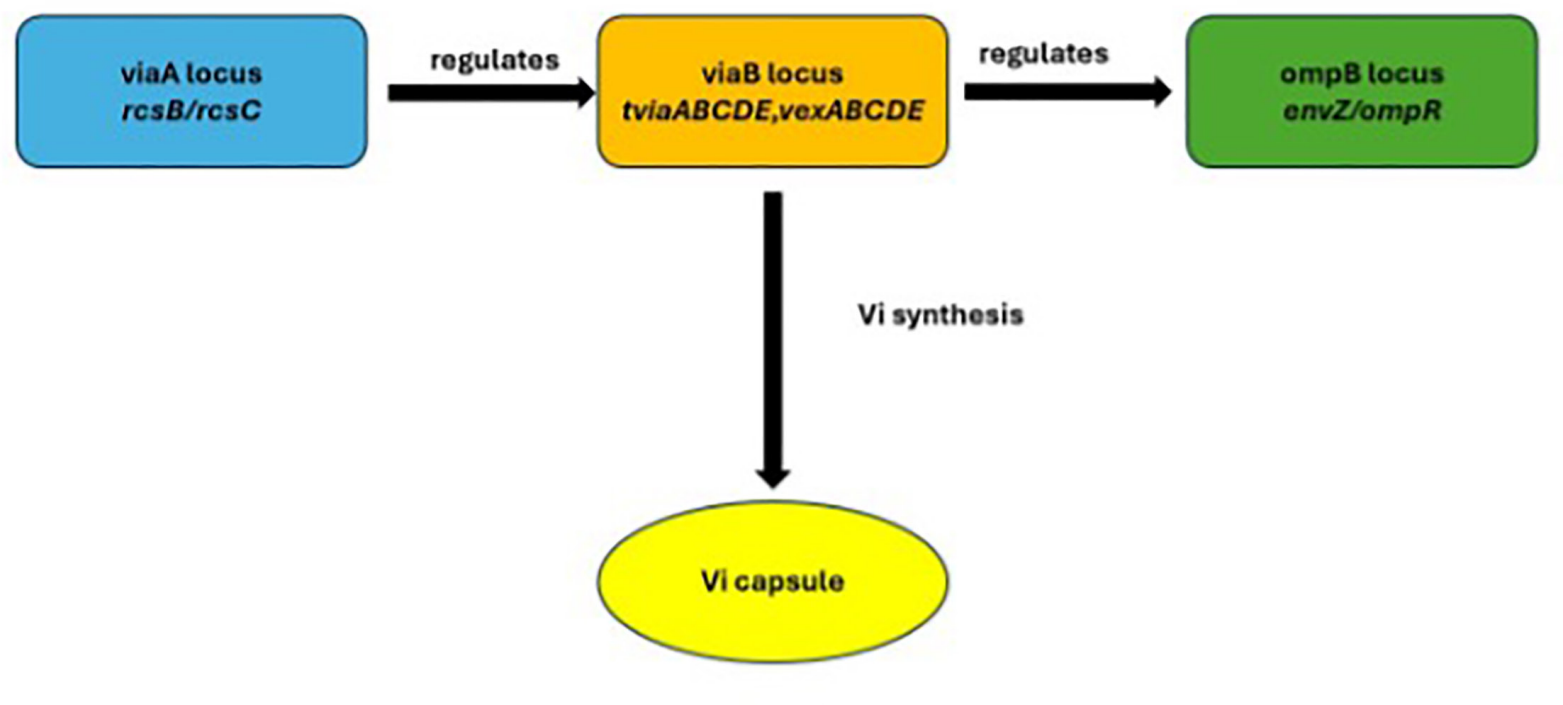
Key genes involved in Vi capsule synthesis included in the analysis.

Raw sequencing reads were processed using the Bactopia pipeline v.3.1.0 [24], which included quality control (https://github.com/s-andrews/FastQC), read trimming, variant calling (https://github.com/tseemann/snippy), AMR gene detection using AMRFinderPlus [25] and lineage assignment (https://github.com/Mykrobe-tools/mykrobe) (`bactopia --samples samples.txt -profile docker --outdir bactopia --use_bakta true --bakta_db /path/to/bakta_db –ask_merlin true`). We used the CT18 reference strain of *S*. Typhi (https://www.ncbi.nlm.nih.gov/nuccore/AL513382.1) as the reference for variant calling via the PHEnix pipeline (https://github.com/ukhsa-collaboration/PHEnix). Resulting consensus sequences were masked for highly variable and repetitive regions [26] using Bedtools v.2.3.0 [27]. A maximum likelihood phylogenetic tree was inferred using IQ-TREE v2.3.5 [28] with in-built model selection (TVM+F+I, best-fit model chosen according to BIC), and tree visualization was performed using iTOL v6.8 [29]. To place our sequencing results into a wider temporal context, we included in the tree the previously sequenced *S*. Typhi isolated from Blantyre (*n*=302) [3] that were not part of the Phase 3 trial (herein referred to as non-trial Blantyre *S*. Typhi isolates). Genes in the viaA regulatory region (represented by *rcsC*), the viaB locus (*tviA–tviE* and *vexA–vexE*) and the OmpB locus (*ompR* and *envZ*), which together regulate Vi capsular polysaccharide expression, were included in the analysis [20,30, 31] (Fig. 1).

To investigate whether H58 isolates contained the IncH1 plasmid that commonly carries the MDR gene cassette associated with H58 *S*. Typhi organisms, PlasmidFinder (Danish Technical University, Denmark: https://cge.food.dtu.dk/services/PlasmidFinder/ was used to detect plasmid presence.

### Statistical analysis

Two-by-two contingency tables were constructed to assess (i) the association between the proportion of isolates with quinolone resistance determining region (QRDR) mutations and vaccine arm, (ii) the association between the proportion of isolates with Vi-capsule mutations and vaccine arm and (iii) the association between the presence of virulence-modifying SNPs and hospitalization among MenA participants. These analyses were performed using the OpenEpi two-by-two module (https://www.openepi.com/TwobyTwo/TwobyTwo.htm).

R version 4.4.1 was used to visualize the distribution of Vi mutations across vaccine arms and to display the association between virulence-modifying SNPs and hospitalization among MenA participants.

## Results

**Fig. 2.**
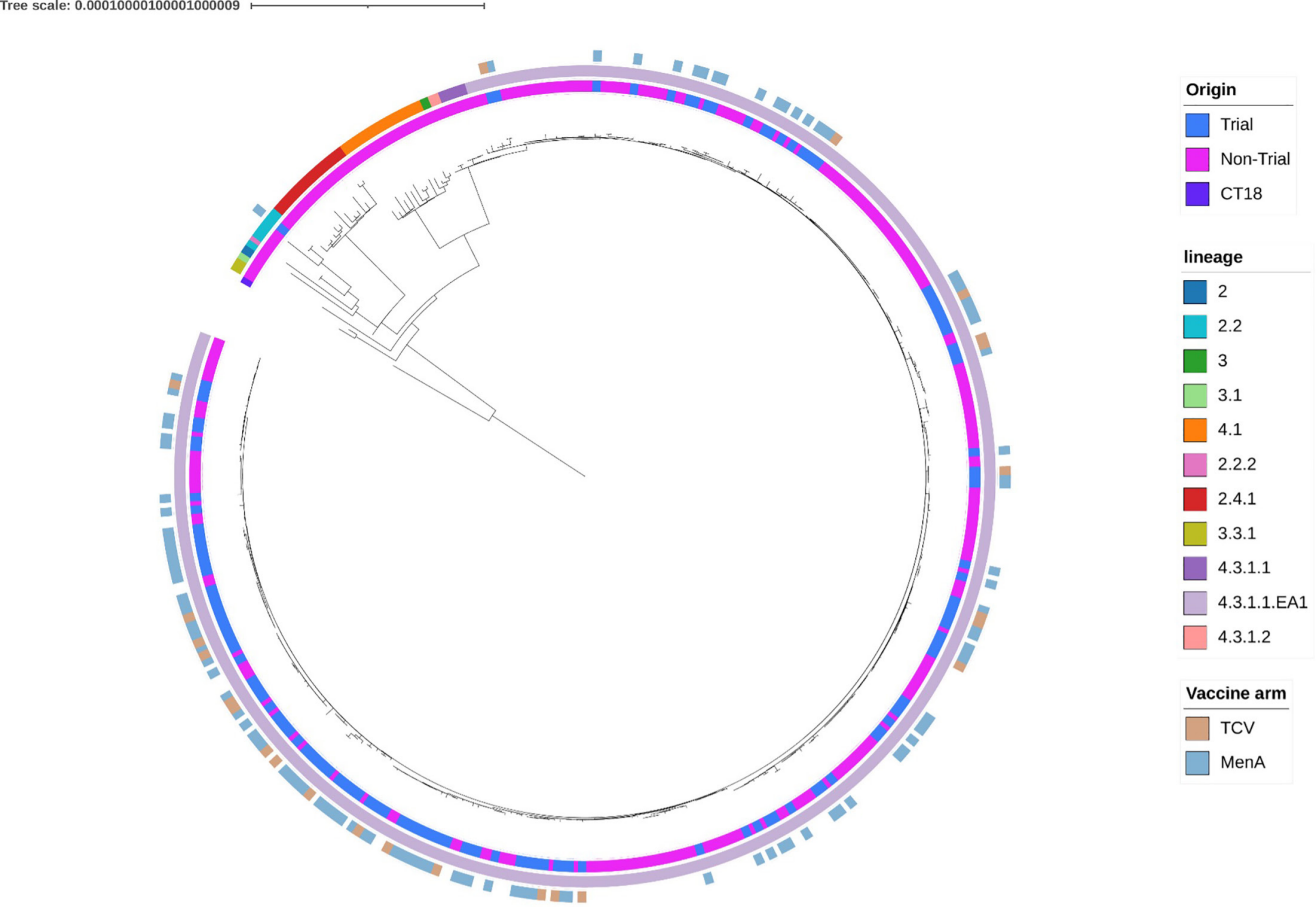
Maximum-likelihood phylogeny of 136 Phase 3 trial isolates contextualized with 302 previously sequenced *S*. Typhi isolates from Blantyre (innermost ring). The middle ring depicts the overall lineage distribution for both the trial and non-trial isolates. The outer ring shows the lineage distribution stratified by vaccine arm for the trial isolates.

Blood culture-confirmed typhoid fever occurred in 134 cases, of which 21 required hospitalization (6 in the TCV arm and 15 in the MenA arm). A total of 136 isolates were sequenced from these cases (Table S1, available in the online Supplementary Material). Two individuals each experienced two distinct clinical episodes of typhoid, one in the TCV arm and one in the MenA arm resulting in two additional isolates. Of these, 135 out of 136 isolates (24 from the TCV arm and 111 from the MenA arm) belong to the L4.3.1.1.EA1 genotype, and the remaining isolate (from the MenA arm) was assigned to lineage 2.2. Importantly, no Vi-negative *S*. Typhi were detected in the trial. In a maximum likelihood phylogenetic tree, no clustering by vaccine arm was observed (Fig. 2). Placing our sequencing results within a broader temporal context shows that H58 (lineage L4.3.1.1.EA1) continues to dominate the *S*. Typhi population in Blantyre (76%, 334 out of 438), with limited genetic diversity observed between the Phase 3 trial isolates and the non-trial Blantyre isolates. PlasmidFinder results suggested that the IncH1 plasmid was not present in any H58 isolate.

### AMR characterization

AMR profiling showed that nearly all sequenced S. Typhi isolates (99%, 135 out of 136) carried genes associated with an MDR phenotype, including *aph(6)-Id*, *aph(3″)-Ib*, *blaTEM-1*, *dfrA7*, *catA1*, *sul1* and *sul2*. Genotypic predictions were fully concordant with phenotypic antimicrobial susceptibility testing (AST), with all 135 MDR isolates demonstrating resistance to ampicillin, chloramphenicol and cotrimoxazole (Table S2). The remaining one isolate lacked MDR-associated genes and was phenotypically pan-susceptible to all tested antimicrobials. The MDR profile distribution was the same across both TCV and MenA arms, with no differences observed (Fisher’s exact test *P*>0.99). Five isolates (3%, 5 out of 136) carried QRDR mutations, including 3 encoding GyrA S83F and 2 encoding GyrB S464F substitutions. Three of these isolates were recovered from the MenA arm and two from the TCV arm with no statistical differences observed (Fisher’s exact test *P*=0.25, Table S4). Based on genotypic predictions, these mutations were associated with intermediate susceptibility to ciprofloxacin; however, AST classified all five isolates as ciprofloxacin susceptible, consistent with reduced fluoroquinolone susceptibility associated with single QRDR mutations in *S*. Typhi.

### Vi locus variants

**Fig. 3.**
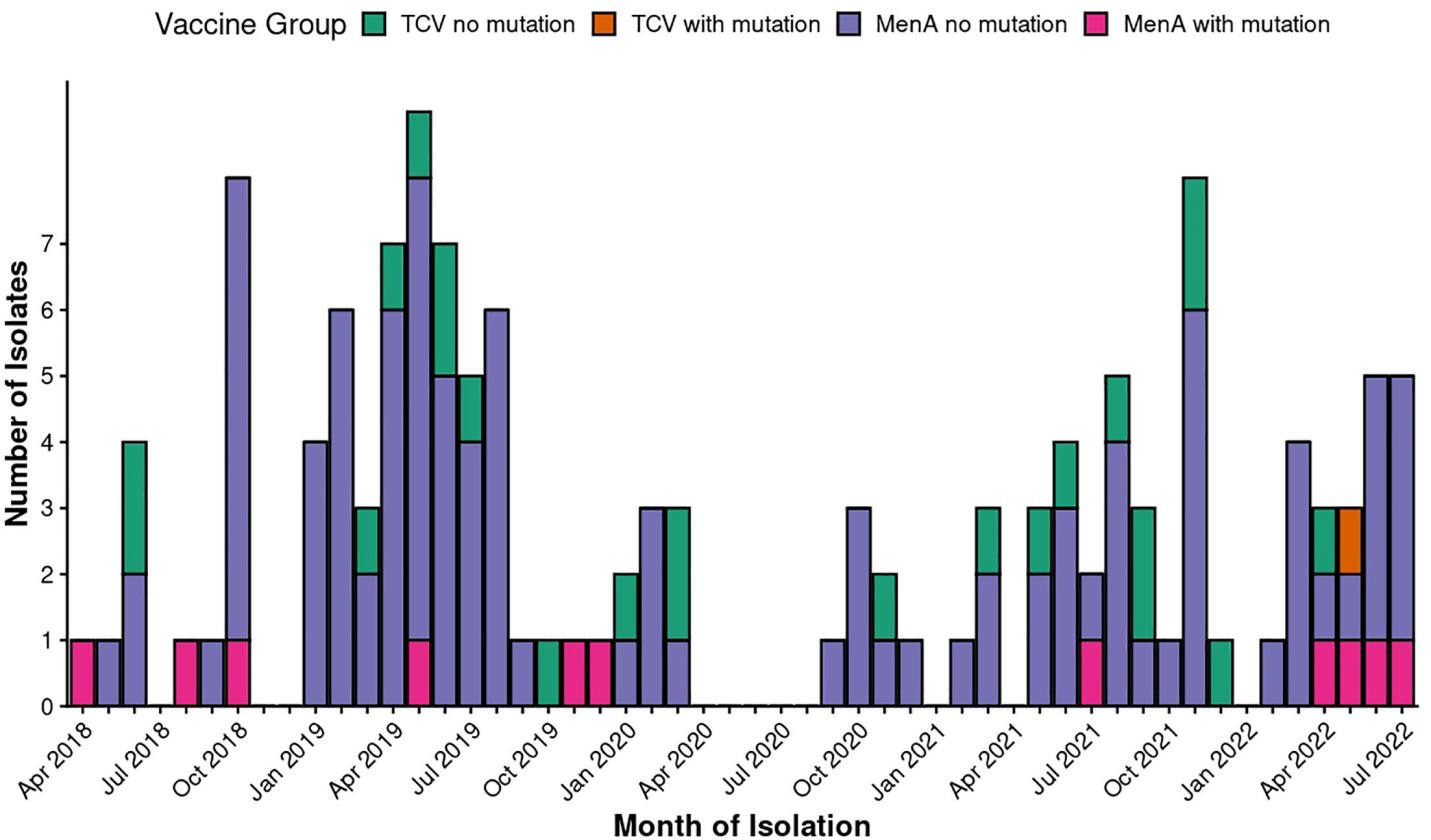
Temporal distribution of blood-culture–confirmed typhoid cases among trial isolates, stratified by vaccine group and presence of mutations within the Vi region. The x-axis represents the month of isolate collection, while the y-axis indicates the number of isolates obtained in each month. Bars are coloured according to four categories: TCV no mutation, TCV with mutation, MenA no mutation and MenA with mutation. ‘TCV’ denotes isolates recovered from participants who received the TCV, whereas ‘MenA’ denotes isolates from participants who received the meningococcal A conjugate vaccine, which served as the control vaccine in the trial. The ‘with mutation’ categories represent isolates in which mutations affecting the Vi region were identified, while the ‘no mutation’ categories represent isolates lacking detectable mutations in this region.

The *S*. Typhi genes encoding the Vi biosynthesis system were present (>=99% of bases covered at >=5× depth) in all isolates. Twelve non-synonymous SNPs in the Vi-capsule encoding genes were identified in 11 of the 136 isolates (Fig. 3, Table S3). Of the 11 isolates, 10 were from the MenA arm, and 1 from the TCV arm (Fig. 3). Trial arm was not statistically associated with the detection of SNPs in Vi capsule encoding genes (Fisher’s exact test *P*=0.67, Table S3). No indels were detected. The SNPs were distributed among the following genes: *tviD* (locus tag STY4659; *n*=6), *tviE* (locus tag STY4656; *n*=5) and *vexA* (locus tag STY4655; *n*=1).

**Fig. 4.**
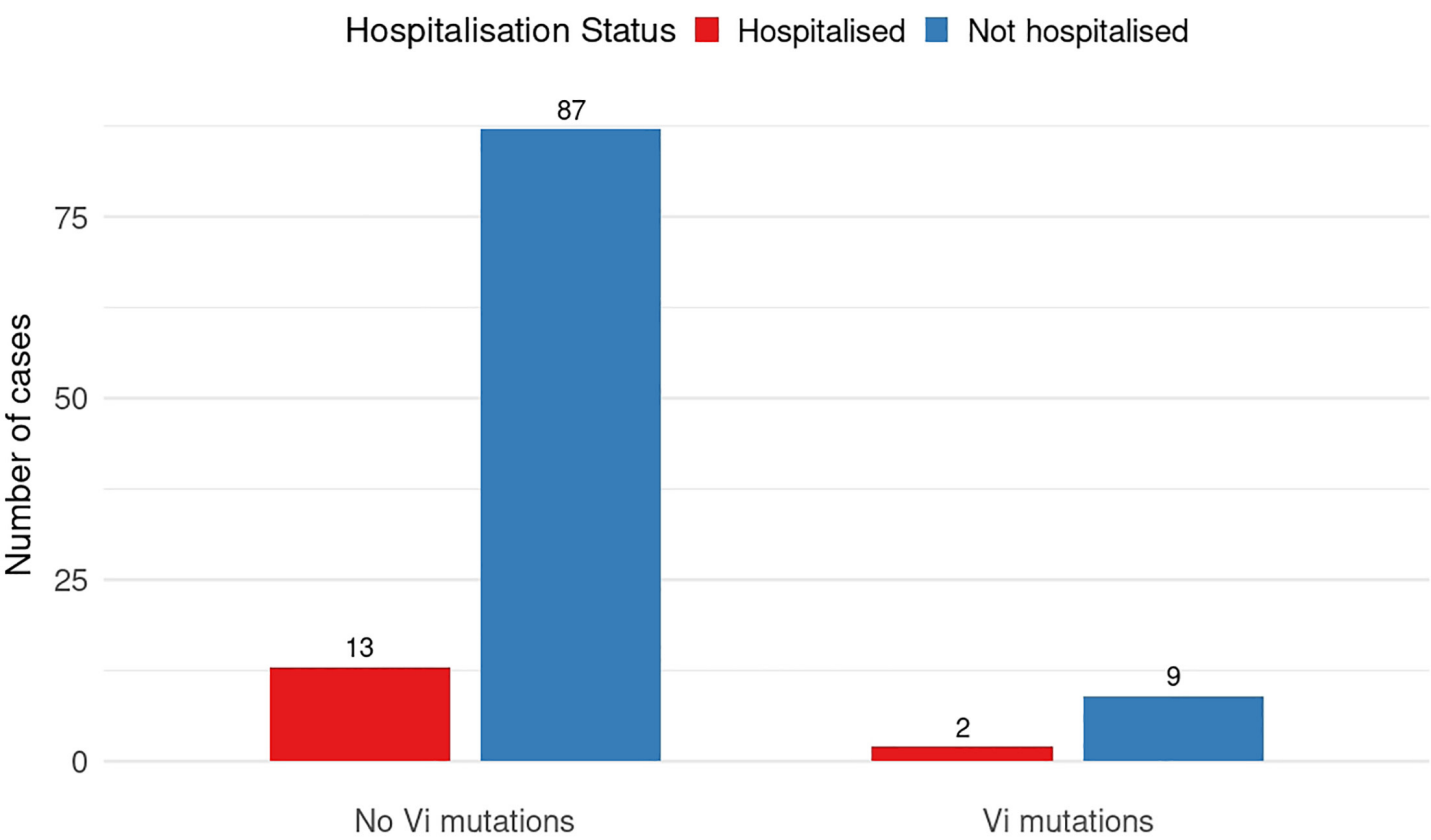
Association between previously reported virulence-enhancing Vi-capsule SNPs and hospitalisation among MenA cases. Fisher’s exact test: *P*>0.91.

Four SNPs encoding amino acid changes – R523H in the TviD protein and Q219R, S290G and C137R in the TviE protein – have previously been reported to increase the virulence of *S*. Typhi infections in mice [20] (Fig. 4). As these SNPs were also identified in our dataset, we analysed their potential association with disease severity in humans, using hospitalization as a proxy for severity (Fig. 4). This analysis was restricted to 112 MenA arm isolates, as the effect of TCV on disease severity in breakthrough infections remains unclear. Of the 111 cases from MenA arm with sequenced *S*. Typhi, 15 were hospitalized. Two hospitalized patients were infected with isolates carrying Vi-capsule SNPs (TviD T11M, TviD A203V and TviE S290G), whereas the remaining 13 hospitalized patients were infected with isolates lacking Vi-capsule SNPs. No association was observed between Vi-capsule SNPs, including previously reported virulence-associated mutations, and hospitalization (Fisher exact test *P*>0.91, Table S4).

### Non-typhoidal *Salmonella*

One potential consequence of introducing a Vi-based vaccine is the emergence of Vi-negative *S*. Typhi, which could be misclassified in our laboratory workflow as non-typhoidal *Salmonella* (NTS). To exclude this possibility, we examined all NTS isolates recovered during the trial. A total of 20 NTS isolates were obtained from children enrolled in the Phase 3 trial. High-quality sequencing data were generated for 17 of these isolates: 9 from the MenA arm and 8 from the TCV arm. Sixteen out of seventeen isolates were *S. enterica* serovar Typhimurium (*S*. Typhimurium), and one was *Salmonella* Bovismorbificans. Among the *S*. Typhimurium isolates, 12 were ST313, 2 were ST19 and 2 were ST3257, which is a single locus variant of ST313. Overall, the following AMR genes were found at most in these NTS isolates: *aadA1*, *aph(3'')-Ib*, *aph(6)-Id*, *blaTEM-1*, *catA1*, *dfrA1*, *sul1* and *sul2*.

## Discussion

In this study, we integrated pathogen whole-genome sequencing into a randomized Phase 3 TCV efficacy trial to test whether vaccination exerted a detectable short-term selective pressure on *Salmonella* Typhi. Four key findings emerged. First, the same MDR L4.3.1.1.EA1 (H58) sub-lineage dominated infections in both trial arms, with no phylogenetic clustering by trial arm. Second, AMR gene content was indistinguishable between vaccine and control arms. Third, non-synonymous Vi-capsule mutations were rare and were not enriched in TCV recipients. Fourth, no Vi-negative *S*. Typhi were detected, either directly or among the NTS isolates that would have captured such organisms in our serotyping workflow. Collectively, these observations provide the first randomized trial genomic baseline against which longer-term, post-introduction surveillance of *S*. Typhi under TCV pressure can be benchmarked.

Our primary hypothesis was that TCV pressure might drive the emergence of Vi antigen variants capable of evading TCV-induced immunity. Although amino acid-altering Vi substitutions were identified, the distribution was similar between the intervention and control arms, which supports the null hypothesis. Our analysis identified no association between four amino acid changes located in the TviD and TviE proteins, previously shown to increase virulence in murine models [20] and disease severity using hospitalization as a proxy. A limitation to both these findings is the small number of isolates from the TCV arm, and the relatively small number of hospitalizations, which limited statistical power. We hypothesized that the reduced antimicrobial use observed in the TCV arm of the trial [22] might translate into fewer AMR genes among *S*. Typhi isolated from TCV recipients. However, this was not supported by our findings. Two factors likely explain this: (1) the dominance of MDR H58 across both vaccine groups, and (2) because AMR genes in Blantyre H58 isolates are chromosomally integrated, the MDR phenotype is expected to be stable regardless of immediate antibiotic pressure [32]. One way in which vaccine escape could manifest is in Vi-negative *S*. Typhi [21], which would be typed as NTS in our laboratory workflow. Therefore, all 20 NTS isolates recovered from blood cultures were genomically confirmed as NTS. The detection of NTS during the trial validates the laboratory serotyping of the trial isolates and confirms the absence of Vi-negative *S*. Typhi in our cases.

To our knowledge, there are no peer-reviewed or preprint reports that present whole genome comparisons of *S*. Typhi isolates stratified by vaccine arm from a randomized TCV trial. Most published genomic work addresses population surveillance or pre/post-introduction comparisons rather than a comparison of trial arms within a randomized study. For example, Thilliez *et al*. conducted a genomic epidemiology study of *S*. Typhi in Harare, Zimbabwe (2012–2019) [33], before the national rollout of the TCV, and da Silva *et al*. studied the population structure and AMR profiles of 174 *S*. Typhi and 54 *S*. Paratyphi A isolates in the context of a phased municipal vaccination campaign in Navi Mumbai, India [34].

Our study has several strengths that enhance its significance. First, it was nested within a clinical trial with good coverage of the study population. Second, genome sequencing of the isolates enabled investigation of multiple genetic aspects, including phylogenetic relationships, AMR determinants and specific SNPs within the Vi biosynthesis region. Finally, the study allowed us to evaluate mutations previously associated with virulence in mouse models within a human clinical context.

However, our study also has limitations. The small number of isolates, especially from vaccinees, limited statistical power to detect subtle vaccine-related genetic differences. Since this study was nested within a clinical trial, we had a relatively brief period of follow-up. The low genetic diversity of *S*. Typhi circulating in Blantyre also limits the power to detect population changes in terms of lineage distribution. Additionally, using hospitalization as a proxy for disease severity does not capture the complete clinical spectrum of typhoid infection. Furthermore, markers of potential hypo- or hypervirulence identified in murine models require validation in larger datasets with clinical metadata and vaccination status. One limitation stems from the slow substitution rate of *S*. Typhi, which compounds the problem of a limited number of isolates and relatively short follow-up time. At a substitution rate of ~0.9 SNPs/genome/year [35,36], the trial follow-up period affords limited phylogenetic resolution for detecting population-level shifts. However, our previous observation that QRDR mutations conferring ciprofloxacin non-susceptibility emerged and increased in frequency within months of rising ciprofloxacin prescriptions in this same Blantyre population [3] demonstrates that *S*. Typhi can respond rapidly to strong selective pressure at the population level. The absence of a comparable signal for Vi-associated mutations in our dataset may reflect fundamental differences in the evolutionary dynamics of vaccine resistance compared with AMR: vaccines act prophylactically and typically target multiple epitopes, generating fewer opportunities for resistance to arise and be selected for than single-target therapeutic drugs [37].

Integration of WGS into vaccine trials in LMIC settings faces several practical barriers that limit the timeliness and scalability of genomic surveillance. Sequencing costs remain high when performed through channel partners, and turnaround times from sample to data can extend to months, making real-time or near-real-time analysis impractical. Containerized bioinformatics workflows such as Bactopia offer reproducibility in principle but depend on reliable, high-bandwidth internet connectivity for downloading container images and reference databases – a requirement that is frequently unmet in the settings where typhoid burden is highest. At present, there are no ready-made solutions to these challenges, and addressing them will require sustained investment in local sequencing infrastructure, offline-capable analysis tools and funding models that treat pathogen genomics as integral to vaccine evaluation rather than as an optional add-on.

No evidence for vaccine escape mutations in this Phase 3 clinical trial setting was detected, but the limitations outlined above mean our findings should be interpreted with caution, and further research with longer follow-up and a larger sample size is required to confirm findings. Given the limited number of participants and follow-up period, continued genomic surveillance in this population after TCV introduction in 2023 will be essential to monitor longer-term evolutionary responses to vaccine pressure in the larger population.

## Conclusion

This study provides an early assessment of *S*. Typhi genomic evolution after TCV introduction in Malawi. Our findings indicate limited divergence between vaccine and control arms, stable AMR profiles and no significant difference in mutations affecting the phenotype of Vi-capsule genes. Continued monitoring of *S*. Typhi populations is essential to track the long-term impact of vaccination on pathogen evolution.

## Supplementary material

10.1099/mgen.0.001721 Supplementary Material 1.

## Abbreviations

AMR: antimicrobial resistance
AST: antimicrobial susceptibility testing
MDR: multidrug-resistant
NTS: non-typhoidal *Salmonella*
QRDR: quinolone resistance determining region
*S*. Typhi: *Salmonella enterica* serovar Typhi
*S.* Typhimurium: *Salmonella enterica* serovar Typhimurium
TCV: typhoid conjugate vaccine
